# Cartilaginous Choristoma of the Oral Cavity: A Rare Presentation in the Nasopharynx

**DOI:** 10.1155/carm/4506082

**Published:** 2024-12-17

**Authors:** Maryam Al-Ali, Anastasios Hantzakos

**Affiliations:** Department of Otolaryngology, Head and Neck Surgery, Cleveland Clinic Abu Dhabi, Abu Dhabi, UAE

**Keywords:** cartilaginous choristoma, nasopharynx, oral cavity, tonsil, tumor

## Abstract

**Objective:** This case report describes a rare presentation of a cartilaginous choristoma of the oral cavity within the tonsillar fossa, emphasizing the importance of recognizing and differentiating this uncommon entity from more frequently encountered oral lesions.

**Methods:** A comprehensive clinical and histopathological examination was conducted on a 30-year-old male patient who presented with a painless mass in the nasopharynx. An excisional biopsy was carried out, and a histopathological analysis was conducted to establish a definitive diagnosis.

**Results:** Histopathological examination demonstrated a cartilaginous choristoma, characterized by the presence of mature hyaline cartilage surrounded by the connective tissue. The patient underwent surgical excision of the lesion, and follow-up assessments indicated a favorable postoperative outcome without recurrence.

**Conclusion:** Cartilaginous choristomas in the oral cavity are exceedingly rare. Awareness of this entity is crucial for accurate diagnosis and appropriate management, as it can mimic other more common oral lesions. This case report contributes to the limited literature on oral cartilaginous choristomas and underscores the significance of considering this entity in the differential diagnosis of oral mucosal masses.

## 1. Introduction

Cartilaginous choristoma is a lesion defined by the presence of mature hyaline cartilage in abnormal locations [1]. While these lesions are more frequently encountered in other anatomical sites, their occurrence within the oral mucosa remains a rarity [2]. This case report explores a distinctive presentation of a cartilaginous choristoma in the oral cavity of a 30-year-old male patient, highlighting the challenges associated with its diagnosis and emphasizing the significance of distinguishing it from other oral lesions.

The oral cavity is a dynamic and complex anatomical region susceptible to a myriad of pathological conditions. Lesions arising from the mesenchymal tissue, such as cartilaginous choristomas, are particularly unusual within the oral mucosa, posing diagnostic dilemmas for clinicians [3]. This report aims to contribute to the limited body of the literature on oral cartilaginous choristomas by detailing the clinical and histopathological features of a singular case, shedding light on its presentation, diagnosis, and management.

Through a thorough examination of the patient's clinical history, surgical and histopathological findings, we aim to provide valuable insights into the distinctive characteristics of cartilaginous choristoma case and we seek to enhance the understanding of oral cartilaginous choristomas, ultimately contributing to a more accurate and hastened diagnosis and improved patient care.

## 2. Case History/Examination

### 2.1. Clinical Presentation

A 30-year-old male, previously healthy, presented with a complaint of nasal obstruction with allergic rhinitis symptomatology. He reports a previous history of adenoidectomy in childhood. He was seen at different facilities prior to his presentation and was told that he has residual adenoids with conflicting opinions regarding removal.

## 3. Methods

**Figure 1:**
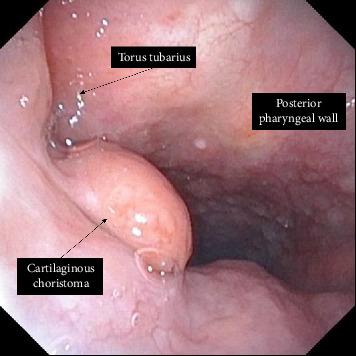
Cartilaginous choristoma of the nasopharynx.

Intraoral examination did not reveal any obvious abnormality. A flexible nasal endoscopy was done which revealed a patent nasopharynx, with a small pedunculated lesion medial to the torus tubarius, and the overlying mucosa appeared intact (as seen in Figure 1). In the presented case, the cartilaginous choristoma was located on the right side of the nasopharynx. Its anatomical boundaries included the posterior aspect of the right nasal cavity, specifically the right choana, anteriorly; the right posterior pharyngeal wall posteriorly; and the midline structures of the nasopharynx, including the pharyngeal raphe, medially. Laterally, the lesion was in close proximity to the right torus tubarius and the pharyngeal orifice of the right eustachian tube. Superiorly, it extended toward the skull base, including the inferior aspect of the sphenoid sinus and the roof of the right nasopharynx, while inferiorly, it was bordered by the right side of the soft palate. There were no palpable lymph nodes in the cervical region. The patient had no relevant medical history or predisposing factors. The patient was asked to come back in 2 months for follow-up.

During the subsequent follow-up, the mass was still present with no change in size or consistency, and the patient did not complain of any new issues. He was then consented for an excisional biopsy of the nasopharyngeal lesion.

### 3.1. Surgical Findings and Removal

An excisional biopsy was performed under general anesthesia. Intraoperatively, the lesion was found to be encapsulated and easily distinguishable from the surrounding tissue, attached to the right posterior pillar with a broad pedicle that was firmly adhered to the underlying tissue. Due to the inaccessible location on the oropharyngeal mucosa, an endoscopic approach was attempted for the lesion's removal but was unsuccessful. Therefore, an intraoral approach was instead used. The mass was palpated on the right posterior tonsillar pillar. It was then exposed by retracting the posterior tonsillar pillar laterally with Blakesley forceps. The mass was grasped with Takahashi forceps. The broad pedicle was cauterized with bipolar diathermy, and the mass was cut with Metzenbaum scissors and sent to pathology in formalin.

The surgical procedure was uneventful, with minimal bleeding, and the patient reported no postoperative complications.

### 3.2. Histopathological Diagnosis

**Figure 2:**
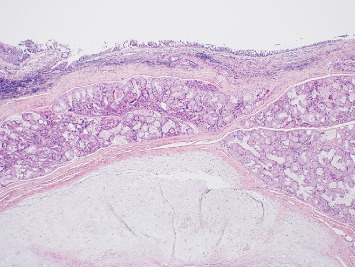
Detailed histopathologic image of a cartilaginous choristoma showing submucosal islands of mature cartilage.

**Figure 3:**
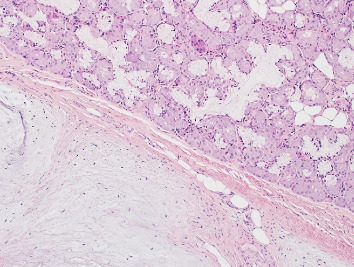
Histopathology of cartilaginous choristoma.

Histopathological examination of the excised specimen revealed a cartilaginous choristoma. The microscopic analysis demonstrated polypoid reactive respiratory type mucosa with submucosal aggregates, and minor salivary glands and submucosal fibrosis. Within the submucosa, there is an island of mature cartilage, which is not present at the tissue margins. No evidence of cellular atypia, mitotic activity, or invasive growth was identified, confirming the benign nature of the lesion (as seen in Figures 2 and 3).

## 4. Results

The patient's postoperative course was unremarkable, with prompt resolution of the initial swelling. Follow-up assessments at regular intervals showed no signs of recurrence. The patient remained asymptomatic, and intraoral examination revealed satisfactory healing of the surgical site.

## 5. Discussion

Cartilaginous choristomas within the oral cavity are exceptionally rare, and their clinical presentation often poses a diagnostic challenge for healthcare professionals.

Berry first described cartilaginous choristomas in 1890. The reported mean age of occurrence is 47 years, with cases documented in individuals ranging from 10 to 80 years of age [4], but it is important to note that the understanding and classification of these anomalies have evolved over time. Choristomas have been reported in various parts of the head and neck region, including the oropharynx, hypopharynx, oral cavity, and middle ear [2]. Oral choristomas most commonly occur in the tongue, with less frequent occurrences reported in other sites within the oral cavity [5].

The clinical presentation of this case aligns with previous reports of oral cartilaginous choristomas, which typically manifest as slow-growing, painless masses without associated symptoms [5].

In this case, a 30-year-old male presented with an asymptomatic oral mass near the tonsillar fossa, ultimately diagnosed as a cartilaginous choristoma through a comprehensive diagnostic approach. It was previously proposed that choristomas situated in the tonsils might be a potential factor contributing to chronic recurrent tonsillitis. Numerous studies have documented the presence of osseocartilaginous choristomas within the palatine tonsil, with approximately 3% of chronic tonsillitis cases showing an association with cartilaginous choristomas based on histopathological examination of specimens obtained after tonsillectomy [6].

The rarity of these lesions necessitates a meticulous diagnostic workup to differentiate them from more common oral lesions such as fibromas, lipomas, or salivary gland tumors.

But the clinical behavior of this condition appears to be that of a benign lesion, with no reported instances of recurrences or malignant transformations [7].

The precise origin of choristoma remains elusive, with some lesions appearing to stem from developmental malformations, while others may have a reactive origin linked to trauma or chronic irritation [7].

Various explanations have been suggested for the occurrence of choristomas, including the following:

1. Arising from cartilaginous embryonic remnants: This theory proposes that the lesion originates from misplaced multipotential cells during development, specifically heterotopic cartilage remnants from the first four branchial arches.
2. Metaplastic transformation of the chondroid tissue: According to this hypothesis, the chondroid tissue undergoes metaplasia, leading to the formation of cartilaginous choristomas.
3. Originating from pluripotent cells: This theory suggests that the choristoma arises from pluripotent cells that are erroneously located in the oral cavity during development.
4. Neoplasm or teratoma with cartilaginous prevalence: This hypothesis proposes a neoplastic or teratomatous origin with a significant presence of cartilage.
5. Mixed salivary gland tumor with cartilaginous predominance: This theory suggests an association with a mixed salivary gland tumor where cartilage is the primary component.

The embryonic rests theory specifically suggests that the lesion originates from heterotopic cartilage remnants from the first four branchial arches. Another potential embryonic origin is remnants of Meckel's cartilage [8].

Surgical excision, the primary mode of treatment for cartilaginous choristomas, was successfully conducted using an intraoral approach in this case.

The absence of postoperative complications and the prompt resolution of the patient's symptoms underscore the efficacy of this approach in managing oral cartilaginous choristomas [4].

Histopathological examination confirmed the diagnosis of a cartilaginous choristoma, revealing the presence of mature hyaline cartilage surrounded by fibrous connective tissues. Importantly, the absence of cellular atypia, mitotic activity, or invasive growth supported the benign nature of the lesion. While cartilaginous choristomas are generally considered benign and do not exhibit malignant transformation, the histopathological examination remains crucial for a definitive diagnosis and to rule out other potential mimicking pathologies [3].

The successful outcome of this case, with no evidence of recurrence during follow-up, aligns with the generally favorable prognosis associated with oral cartilaginous choristomas following complete surgical excision. Nonetheless, the rarity of these lesions emphasizes the importance of reporting individual cases to contribute to the limited body of the literature, enhancing our collective understanding of their clinical behavior and optimal management.

## 6. Conclusion

In conclusion, this case report highlights the significance of a comprehensive diagnostic and therapeutic approach in managing rare oral lesions such as cartilaginous choristomas. The successful intraoral removal and favorable postoperative course support the efficacy of this technique, while the histopathological analysis remains pivotal for an accurate diagnosis and subsequent patient care. Continuous reporting of such cases will contribute to a more nuanced understanding of oral cartilaginous choristomas and aid in refining diagnostic and therapeutic strategies for these uncommon entities [9].

## Data Availability

The data that support the findings of this case report are included within the article. Additional clinical details and anonymized patient data are available upon reasonable request from the corresponding author, subject to compliance with ethical guidelines and institutional policies. To ensure patient privacy, raw data cannot be publicly shared.
